# Pathologic function and therapeutic potential of extracellular vesicle miRNA in sepsis

**DOI:** 10.3389/fphar.2024.1452276

**Published:** 2024-12-18

**Authors:** Rou Deng, Xiayu Cui, Runze Zhang, Changya Liu, Jielian Luo, Liang Liu, Wen Zhang, Bangjiang Fang

**Affiliations:** ^1^ Department of Emergency, Longhua Hospital, Shanghai University of Traditional Chinese Medicine, Shanghai, China; ^2^ Shaanxi University of Chinese Medicine, Xianyang, China; ^3^ Institute of Emergency and Critical Care Medicine, Shanghai University of Traditional Chinese Medicine, Shanghai, China

**Keywords:** extracellular vesicle, microRNA, sepsis, programmed cell death, inflammation, endothelial dysfunction, therapy, diagnosis

## Abstract

Sepsis is a systemic inflammatory response initiated by an infection, which can lead to multi-organ dysfunction. The pathophysiology of sepsis is complex, and treatment options are limited. Traditional antibiotic therapies have shown limitations, such as promoting the emergence of antibiotic-resistant bacteria and disrupting the natural microbiota. Consequently, there is a pressing need to explore diverse therapeutic approaches for sepsis management. Extracellular vesicles, which play a crucial role in cell-to-cell communication, are released by various cell types throughout the body and possess a membrane structure composed of a lipid bilayer. MicroRNAs may be encapsulated within these structures and can be selectively delivered to target recipient cells through the activation of cell surface receptors or via endocytosis and fusion, thereby modulating the biological functions of target cells. The article examines the pathological alterations that happen as sepsis progresses and the biological control of extracellular vesicles and microRNAs in sepsis. This review focuses on the role of extracellular vesicles and their microRNAs on controlling the inflammatory response, macrophage polarization, programmed cell death, endothelial dysfunction, and microcirculatory changes in sepsis. Furthermore, the obstacles encountered by this novel therapy are also examined.

## 1 Introduction

Sepsis is a complex and heterogeneous syndrome caused by a dysregulated host of responses to infection (Levy et al., 2003; Rudd et al., 2020). Sepsis represents a significant global health burden, contributing to elevated rates of morbidity and mortality. Current treatment strategies primarily emphasize antibiotic therapy and symptomatic management, revealing a deficiency in effective targeted treatment approaches (Rudd et al., 2018; 2020; Jarczak et al., 2021). Furthermore, the absence of rapid and reliable diagnostic tools complicates accurate diagnosis. This uncertainty may result in delayed diagnosis or overtreatment. Extracellular vesicles (EVs) are a class of vesicles with a membrane structure and are released by cells. An increasing number of studies have demonstrated that EVs serve as carriers of genetic material, transporting signaling molecules to both nearby and distant cells. This capability enables them to modulate cellular responses that contribute to the onset and progression of various major diseases (Guillaume et al., 2018). Studies have demonstrated that EVs derived from mesenchymal stem cells, tumor cells, T cells, and B cells are rich in microRNAs (miRNAs), indicating that miRNAs are among the primary components transported by EVs (Mittelbrunn et al., 2011; Melo et al., 2014; Asgarpour et al., 2020; Xiong et al., 2023). MiRNAs are a class of non-coding RNA that regulates gene expression by binding to several sections of mRNA, including the coding sequence, 3′untranslated region (UTR), 5′UTR, and gene promoter. This interaction either results in translation inhibition or mRNA destruction (Younger and Corey, 2011) Studies have demonstrated that EVs from patients with septic shock exhibit distinct miRNA expression profiles at different stages of the illness (Azevedo et al., 2013). The observed shift in specificity suggests that the miRNAs carried out in EVs may serve as promising biomarkers and paracrine messengers for communication within and between organs, as well as potential therapeutic targets. This review provides a comprehensive examination of the role of miRNAs associated with extracellular vesicles (EV-miRNAs) in intercellular and inter-organ communication during sepsis progression. Additionally, it analyzes the challenges associated with utilizing and targeting EV-miRNAs as potential diagnostic biomarkers and therapeutic agents. Furthermore, we highlight recent studies that elucidate the mechanisms underlying the specific incorporation of miRNAs into EVs.

## 2 Extracellular vesicles and the miRNA in extracellular vesicles

### 2.1 Extracellular vesicles

**FIGURE 1:**
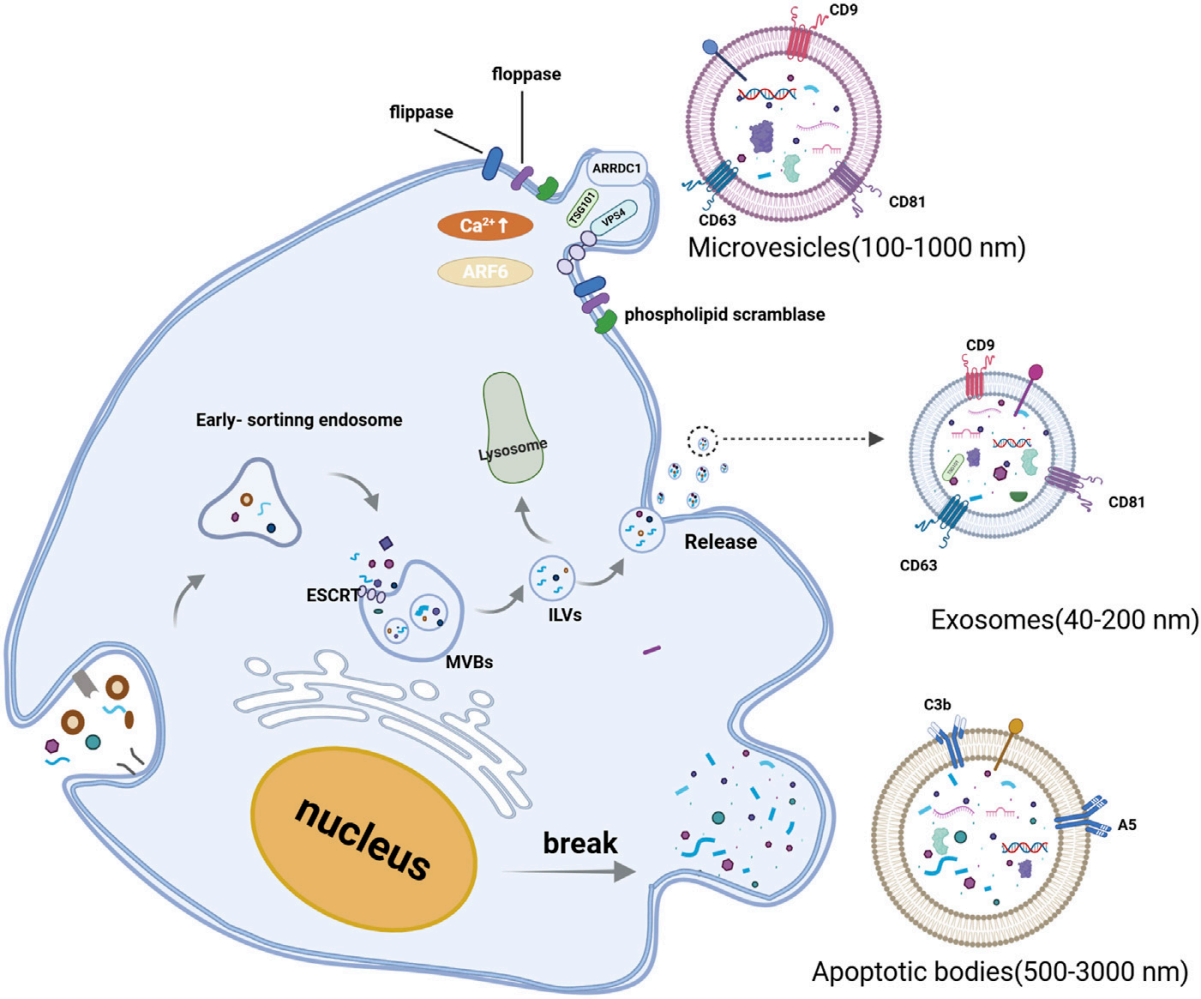
Biogenesis and classification of extracellular vesicles. Extracellular vesicle includes exosomes, microvesicles, and apoptotic bodies. Exosomes, are generated within the endosomal system. Early sorting endosomes can invaginate to form multivesicular bodies (MVBs), which contain intraluminal vesicles (ILVs). MVBs can either fuse with lysosomes for degradation or merge with the plasma membrane to release ILVs into the extracellular space. Microvesicles are formed at the cell surface through outward budding of the plasma membrane. When the nucleus is broken, the cell membrane protrudes outwards, wrapping the fragments of the organelles to form apoptotic bodies. (Created with BioRender.com).

EVs are primarily categorized into three groups based on their size, biological characteristics, and formation processes: exosomes, microvesicles, and apoptotic bodies (Shao et al., 2018). Exosomes originate from the invagination of the cell membrane, which undergoes a secondary invagination to form intracellular multivesicular bodies (MVBs). Upon fusion of MVBs with the cell membrane, the intraluminal vesicles (ILVs) are released as exosomes into the extracellular space. Exosomes typically range in diameter from 40 to 200 nm. Microvesicles are generated through the budding of the cell membrane, with diameters ranging from 200 to 1,000 nm. Apoptotic bodies are membrane-wrapped vesicles that have a diameter of 500–3,000 nm and contain organelles and cytoplasm. They are formed during apoptosis and cellular atrophy (Cocucci et al., 2009; Raposo and Stoorvogel, 2013; Rädler et al., 2023) (see Figure 1). Both microvesicles and exosomes can transport mRNA, miRNA, and proteins to recipient cells, thereby facilitating intercellular information transfer. The formation of microvesicles is associated with cytosolic calcium concentration and the presence of arrestin domain-containing protein 1 (ARRDC1). On one hand, Ca2+-dependent proteins, such as phospholipid scramblase, degrade the membrane-bound cytoskeleton to promote budding. On the other hand, ARRDC1 recruits ESCRT, TSG101, and VPS4 proteins to the membrane to facilitate budding (Abels and Breakefield, 2016; Shao et al., 2018). Apoptotic bodies are formed by the random blebbing of the cell membrane, which is a consequence of cell death (Andaloussi et al., 2013; Guillaume et al., 2018; Gurunathan et al., 2019). The diameters of exosomes, microvesicles, and apoptotic bodies have varied widely in the literature over the past decade. Several studies indicate that the diameter of apoptotic bodies ranges from 50 nm to 4,000 nm (Akers et al., 2013). This overlap in size distribution can complicate the isolation of exosomes, as apoptotic bodies may co-purify with exosomal preparations. The Minimal Information for Studies of Extracellular Vesicles 2023 (MISEV2023) continue to caution against using terms such as “exosomes” and “microvesicles,” which are often classified size without considering their biogenesis (Welsh et al., 2024). As with MISEV2018, MISEV2023 has not yet specified universal markers for each EV subtype. Notably, MISEV2023 emphasizes the use of orthogonal methods (including imaging, particle tracking, and biochemical assays) to increase the confidence in EV characterization, rather than accurately characterizing EVs by a single molecular marker (Théry et al., 2018; Welsh et al., 2024).

A growing number of researchers are focusing their attention on the potential role of EVs in sepsis. In the past few years, many studies have shown that sepsis patients have higher amounts of EVs. These EVs are isolated from plasma, urine, red blood cells, various immune cells, and endothelial cells (Soriano et al., 2005; Zhang et al., 2016). Furthermore, Azevedo et al. have identified that platelet-derived exosomes in septic shock patients may contribute to cardiac dysfunction (Azevedo et al., 2007). Most research suggest that EVs transport proteins, lipids, and nucleic acids via endocytosis and pinocytosis, resulting in biological responses. Since 2013, the Vesiclepedia database has recorded 22,858 EV-miRNAs, and this number continues to grow, reflecting an increasing recognition of the unique functions of EV-miRNAs in biological systems. This review aims to elucidate the role of EV-miRNAs in the pathophysiological processes of sepsis and to explore their potential as diagnostic and therapeutic tools for this condition.

### 2.2 miRNA biogenesis

**FIGURE 2:**
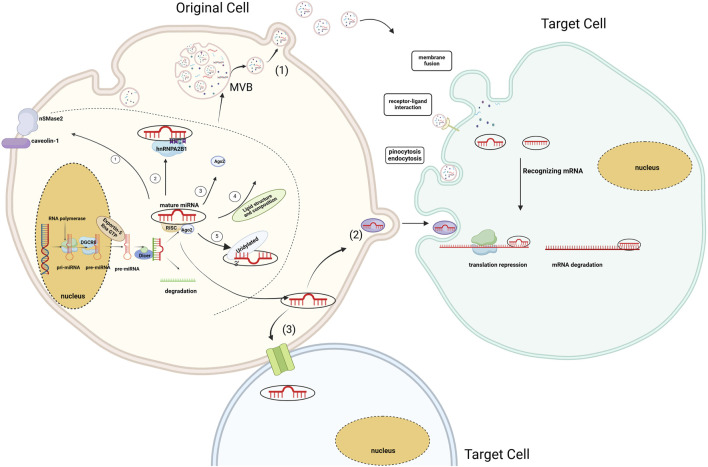
Biogenesis of miRNA and EV-miRNA sorting mechanism. MiRNAs sort into EVs through five main pathways ① Membrane Protein-Associated Pathway; ② RNA-Binding Protein and Specific Motif Pathway; ③ miRISC-Associated Pathway; ④Associated with Lipid Composition, Structure; ⑤ Specific sequences or modifications at the 3′end of miRNAs. MiRNAs are transported to recipient cells in three ways (1) Transport of miRNAs via extracellular vesicles (2) Transport of miRNAs via RNA-binding proteins (3) Direct transfer to neighbouring cells via gap junctions formed by connexins (Hong et al., 2015). (Created with BioRender.com).

MiRNA, a class of non-coding RNA, consists of 18–25 nucleotides and are ubiquitously present in eukaryotes. They typically originate in the introns of protein-coding genes. These genes are transcribed into primary miRNAs (pri-miRNAs), characterized by a stem-loop structure, by RNA polymerases II and III. Pri-miRNAs undergo a two-step cleavage process to yield mature miRNAs. First, in the nucleus, pri-miRNAs are processed into precursor miRNAs (approximately 60–70 nucleotides) with the assistance of Drosha and DGCR8. These precursor miRNAs are then exported to the cytoplasm by Exportin-5 and Ran GTP, where Dicer performs further cleavage (Lee et al., 2003; Gregory et al., 2004; Rie et al., 2017). The second step of cleavage occurs in the cytoplasm. Dicer is generally believed to determine the cleavage position through the mechanism known as the “molecular ruler.” The Dicer enzyme recognizes pre-miRNAs and generates two strands of a length of about 22 bases with the help of the PAZ-like domain and the RNase III domain (Li and Patel, 2016; Leitão and Enguita, 2022). However, a recent study found a deeply conserved cis-acting element located near the cleavage site of pre-miRNAs, termed the GYM motif. This motif is recognized by the dsRBD domain of Dicer, enhancing Dicer’s efficiency and precision in cleaving pre-miRNAs. Thus, the GYM motif is regarded as a key element in ensuring the accuracy of Dicer’s function in miRNA processing (Lee et al., 2023). Subsequently, with the assistance of the Argonaute (Ago) protein, one strand preferentially binds to the RNA-induced silencing complex (RISC) to generate the mature miRNA (Chendrimada et al., 2005; Maniataki and Mourelatos, 2005) (see Figure 2).

It is generally believed that miRNA can recognize binding sites on the 3′UTR of target gene mRNA by using the seed sequence (nucleotides 2–8 at the 5′end), leading to mRNA degradation or inhibition of mRNA translation, ultimately resulting in gene silencing (Lewis et al., 2003). However, recent studies suggest that mammalian miRNAs may facilitate the deadenylation and decapping of target mRNAs by recruiting deadenylase complexes, including the PAN2-PAN3 complex and the CCR4-NOT complex. Subsequently, the target mRNA is degraded by the cytoplasmic enzyme 5′-to-3′ exoribonuclease 1 (XRN1) (Jonas and Izaurralde, 2015; Kobayashi and Tomari, 2016). In addition, miRNAs can also act as regulators of a variety of biological processes by binding to functional proteins, directly activating TLR receptor proteins, regulating mitochondrial mRNA, and targeting precursor RNAs that negatively regulate other non-coding RNAs (Barrey et al., 2011; Lehmann et al., 2012; Tang et al., 2012; Meister, 2013). MiRNAs play a role in various biological processes and diseases, including sepsis in Chen et al. observed that miR-133a, a regulator of macrophages, was significantly upregulated in septic patients and in cecum ligation (CLP) mouse models, while miR-29b-3p and miR-96-5p were downregulated (Chen et al., 2020; Formosa et al., 2022). However, isolated miRNAs are highly susceptible to degradation by nucleases. Therefore, they are typically associated with RNA-binding proteins such as AGO2, or high-density lipoprotein, or encapsulated within EVs such as exosomes for stability and effective transport (Turchinovich et al., 2016). Here, we focus on the role of EV as a miRNA carrier.

### 2.3 The mechanism of miRNA entering extracellular vesicles

EVs containing miRNAs are associated with the onset and progression of numerous diseases. Their functions encompass gene regulation and intercellular communication. EVs can selectively sort miRNAs, suggesting that alterations in EV-miRNAs may influence disease progression. Recent investigations have shown that Ago2, a critical component of the RISC, binds miRNAs and facilitates their incorporation into EVs. Ago2 regulates the entry of various of miRNAs into exosomes, such as let-7a, miR-100, miR-320a, miR-451, miR-150 and miR-142-3p (Guduric-Fuchs et al., 2012; McKenzie et al., 2016). Moreover, membrane- associated proteins like caveolin-1 and the neural Sphingomyelinase 2 (nSMase2) are linked to EV-miRNA classification (Groot and Lee, 2020). The first EV-miRNA sorting protein, nSMase2, regulates EV formation by producing ceramide in the plasma membrane and endosomes. Overexpression or knockdown of nSMase2 controls EV-miRNA levels (Kosaka et al., 2010). Subsequent studies have shown that the levels of exosomal miRNAs (such as miR-202, miR-16, miR-29b, and miR-15) have significantly changed in MM cells treated with ceramide C6. These findings suggest that ceramide plays a role in the sorting of exosomal miRNAs (Cheng et al., 2018). Moreover, specific motifs of miRNAs are regarded as crucial factors in the EV-miRNA sorting process. On one hand, EVs are formed by the spontaneous inward budding of the raft region of the MVB membrane. Unique nucleotide sequences exhibit different affinities for the lipid bilayer. Consequently, miRNAs with particular nucleotide sequences preferentially associate with lipid raft domains (Janas et al., 2015). On the other hand, miRNA-specific motifs are typically recognized as specific RNA-binding proteins and incorporated into EVs. Carolina et al. were the first to confirm that sumoylated hnRNPA2B1 in primary T lymphoblasts can identify the GGAG motif of miR-198 and miR-601, thereby regulating their transport into exosomes (Villarroya-Beltri et al., 2013). Over the subsequent years, various RNA-binding proteins, including Y-box binding protein 1 (YBX1), Lupus La, and KRAS, were discovered to have a strong association with the selection of exosomal miRNAs (Cha et al., 2015; Shurtleff et al., 2016; Statello et al., 2018; Temoche-Diaz et al., 2019). (More information of RNA-binding proteins Table 1). Furthermore, studies have shown that 3′end adenylated miRNAs are relatively enriched in cells. In contrast, 3′end uridylated miRNAs have a higher expression level in EVs, suggesting that post-transcriptional modification of miRNA may be a possible mechanism guiding miRNA sorting into EVs (Koppers-Lalic et al., 2014) (see Figure 2).

**TABLE 1:** Compilation of RNA-binding proteins (RNPs) participating in the sorting of microRNA (miRNA) within extracellular vesicles.

RNA binding proteins	Related miRNA	Recognition sequence	Ref.
hnRNPA2B1	miR-198 and miR-601	GGAG	Villarroya-Beltri et al. (2013)
hnRNPA1	miR-483-5p	—	Liu et al. (2021a)
hnRNPU	miR-221	CAUUG	Liao et al. (2023)
SYNCRIP	miR-3470a and miR-194-2-3p	GGCU	Santangelo et al. (2016)
YBX1 and YBAP1	miR-223	UCAGU	Shurtleff et al. (2016), Liu et al. (2023)
MVP	miR-193 a	—	Teng et al. (2017)
HuR	miR-122	—	Mukherjee et al. (2016)
MEX3C	miR-451a	—	Lu et al. (2017)
La protein	miR122	UUU/UGGA	Temoche-Diaz et al. (2019)
Alyref	—	CGGGAG	Garcia-Martin et al. (2022)
FUS	miR-483-5p	CGGGAG	Garcia-Martin et al. (2022), Gordillo Sampedro et al. (2023)
SRSF7	—	—	Gordillo Sampedro et al. (2023)
CELF5	—	—	Gordillo Sampedro et al. (2023)
FMR1	miR-155	AAUGC	Wozniak et al. (2020)

Abbreviation: hnRNPA1, heterogeneous nuclear ribonucleoprotein A1; hnRNPA2B1, heterogeneous nuclear ribonucleoprotein A2B1; SYNCRIP, synaptotagmin-binding cytoplasmic RNA-interacting protein; YBX1, Y-box binding protein 1; MVP, major vault protein; HuR, human ELAV, protein HuR; MEX3C, mex-3 RNA, Binding Family Member C; la protein, Lupus La protein; Alyref, Aly/REF, export factor; FUS, fused in sarcoma; SRSF7, Serine/Arginine rich splicing factor family 7; CELF5,CUGBP, Elav Like Family Member 5; FMR1, Fragile X mental retardation protein; “—”,The EXOmotif and/or regulated miRNAs, were not reported.

## 3 EV-miRNAs in the pathogenesis of sepsis

EVs mediate intercellular communication through various mechanisms, including endocytosis, receptor-ligand interactions, and direct fusion with the plasma membrane (Mulcahy et al., 2014). Considering the capacity of EVs to communicate both locally and distally, it is logical to anticipate that EVs play a role in the development of sepsis. The role of EVs depends on the composition of their contents. MiRNAs can be transported via EVs, and it plays important roles in regulating immune cell function, bioenergetics and metabolism (Ying, et al., 2017; Zhou J. et al., 2018; Diaz-Garrido et al., 2022) Numerous clinical studies have demonstrated that septic patients exhibit distinct types and abundances of miRNAs compared to healthy individuals. For example, Kadri et al. extracted urinary exosomes to identify sepsis-associated miRNAs in comparison to the control group. They found that 94 miRNAs were dysregulated in septic patients, with 29 of them showing highly correlated with the sepsis group (Kadri et al., 2018). Moreover, Juliana et al. compared the miRNAs carried by EVs isolated from the serum of healthy volunteers and sepsis patients. Compared to the control group, 65 miRNAs were differentially expressed, with 28 maintaining their differential expression over a period of 7 days. These miRNAs were found to be associated with pathogenic pathways such as inflammatory response, oxidative stress, and cell cycle regulation (Real et al., 2018). Therefore, EV-miRNAs may be one of the potential mechanisms of the occurrence and development of sepsis (Table 2).

**TABLE 2:** The role of EV-miRNAs in the development of sepsis.

Pathological phenotype	miRNA	Regulation in sepsis	Sample source	Target cell	Potential functional mechanism	Ref.
Regulation of Immune Response	miR-155	Up	Serum	Macrophages	Targeting SHIP1 and SOCS1 to promote macrophage proliferation and inflammation	Jiang et al. (2019)
	miR-155-5p	Up	Stimulated macrophages	Macrophages	Downregulating of MSK1 expression and activating the p38/MAPK pathway	Xu et al. (2023)
	miR-30d-5p	Up	Polymorphonuclear neutrophils	Macrophages	Targeting SOCS-1 and SIRT1 and activating the NF-κB pathway	Jiao et al. (2021)
	miR-19b-3p	Up	Tubular epithelial cells	Macrophages	Targeting SOCS-1 and activating the NF-κB pathway	Lv et al. (2020)
	miR99a/b	Up	Endothelial cells	Monocyte	Altering the expression of monocyte surface CD11b and MHCⅡ	Fitzpatrick et al. (2022)
	miR-1298-5p	Up	Serum	BEAS-2B	Targeting SOCS6 via STAT3 pathway to increased cell permeability and inflammatory response	Ma et al. (2021)
Programmed Cell Death	hsa-miR-1262	Up	Serum	Myocardial cell	Silencing of SLC2A1 promoted apoptosis and suppressed glycolysis in cells	Sun et al. (2022a)
	miR-885-5p	Up	Serum	Myocardial cell	Targeting HMBOX1 and activating the NF-κB pathway	Tu et al. (2022)
	miR-93-5p	Up	Macrophages	Renal epithelial cells	Targeting TXNIP to mediate the pyroptosis of renal epithelial cells	Juan et al. (2021)
	miR-210-3p	Up	Plasma	BEAS-2B	Targeting ATG7 to inhibit the effect of ATG7 on autophagy, promoting cell apoptosis	Li et al. (2021)
	MiR-223-3p	Down	Plasma	Human coronary artery endothelial cells	Negatively regulates NLRP3-dependent inflammasome to suppress pyroptosis in endothelial cells	Wan et al. (2024)
Endothelial dysfunction and microcirculatory alterations	miR-15b-5p and miR-378a-3p	Up	Platelet	Neutrophil	Targeting PDK1 to modulate the Akt/mTOR pathway and then promote NET formation	Jiao et al. (2020)
	miR-1-3p	Up	Plasma	HUVECs	Targeting SERP1 inhibits cell proliferation, and increases monolayer endothelial cell permeability and membrane injury	Gao et al. (2021)
	miRNA-155 and miR-146a	Up	Broncho-alveolar lavage	Bronchial epithelial cells	Not described	Yuan et al. (2018)
	miR-223	up	Platelet	HCAECs	Downregulating ICAM1 expression	Szilágyi et al. (2021)

Abbreviation: SHIP1, Src homology 2 domain-containing inositol-5-phosphatase 1; SOCS1, Suppressor of Cytokine Signaling 1; MSK1, mitogen- and stress-activated protein kinase-1; SIRT1, sirtuin 1; NF-κB, nuclear factor κB; MHCⅡ, Major Histocompatibility Complex class II; SOCS6, Suppressor of Cytokine Signaling 6; STAT3, Signal Transducer and Activator of Transcription 3; SLC2A1, Solute Carrier Family 2 Member 1; HMBOX1, Homeobox Containing 1; TXNIP, Thioredoxin Interacting Protein; ATG7, Autophagy Related 7; NLRP3, NOD-, LRR- and, pyrin domain-containing protein 3; PDK1, Pyruvate Dehydrogenase Kinase 1; NET, neutrophil extracellular trap; SERP1, Stress-associated Endoplasmic Reticulum Protein 1; ICAM1, Intercellular Adhesion Molecule 1.

### 3.1 Macrophage polarization

The role of EV-miRNAs in regulating immune cell function has long captivated researchers. Zhou et al. demonstrated that tumor-associated macrophage-derived exosomes enriched in two miRNAs, specifically miR-29a-3p and miR-21-5p, directly inhibited STAT3 and regulated Treg/Th17 cells, thereby modulating the tumor immune microenvironment (Zhou J. et al., 2018). Liu et al. found that the levels of small extracellular vesicles (sEVs) MIR497HG, miR-195, miR-497, and PD-L1 in sepsis patients fluctuated across distinct stages of immunoactivation and immunosuppression. They hypothesized that the phenomena was related to T-lymphocyte apoptosis. However, the intricate mechanisms underlying these EV-miRNAs in sepsis remain unclear (Liu et al., 2023). Garrido e al. have found that when dendritic cells (DCs) are stimulated by EVs separated from various strains, the EVs released by these DCs have variable miRNA expression. The differential expression of these miRNAs primarily influenced the production of Th1- and Treg-specific cytokines (Diaz-Garrido et al., 2022). Sepsis is caused by infection, and when pathogenic microorganisms invade, macrophages, as important cells in the intrinsic immune system, play a crucial role in the development of sepsis through their polarization, autophagy, and regulation of the inflammatory response. We focused on the involvement of EV-miRNA in macrophage polarization. Typically, macrophage polarization results in two different phenotypes: M1 type for classical activation and M2 type for alternative activation, which is determined by the surrounding microenvironment. M1 macrophages are known for producing high levels of pro-inflammatory substances, such as TNF-α, IL-1β, IL-6, and reactive oxygen species, linking them to inflammatory responses. In contrast, M2 macrophages display an ability to release anti-inflammatory cytokines such as IL-4, and IL-10 (Patel et al., 2017; Chen X. et al., 2021). Although the pro-inflammatory M1 macrophages have strong capabilities in killing microorganisms and anti-tumor, excessive pro-inflammatory responses may contribute to damage to surrounding normal tissues and organs. Similarly, while anti-inflammatory M2 macrophages play a crucial role in suppressing inflammation and promoting recovery, excessive anti-inflammatory responses may also have adverse effects. There is literature highlighting the importance of macrophage polarization in the pathological process of sepsis (Jin et al., 2022). Multiple studies have shown that the EV function as transport mediators, delivering particular miRNAs to promote macrophage polarization. Jiang et al. confirmed that miR-155 was one of the most enriched miRNAs in serum EVs during infection and was transported into macrophages. This miRNA promotes the formation of M1 macrophages and induces robust cytokines production by targeting SHIP1 and SOCS1 during sepsis-related acute lung injury (Jiang et al., 2019). Furthermore, Xu et al. also found that EVs produced by macrophages stimulated by hypervirulent *Klebsiella pneumoniae* could activate resting macrophages, resulting in a significant number of activated macrophages. This process is mediated by EV-enriched miR-155-5p, which targets the MSK1/DUSP1 axis to activate the p38-MAPK signaling pathway. This ultimately leads to M1 macrophage polarization and inflammatory responses (Xu et al., 2023). Both studies indicate that EV-enriched miR-155 and miR-155-5p have a similar effect on macrophage polarization, indicating that these miRNAs could be potential targets for clinical intervention in sepsis. A recent study found that TNF-α-activated neutrophils release pro-inflammatory EVs during sepsis. These EVs transfer miR-30d-5p to macrophages, promoting their polarization into M1 macrophages. This miRNA activates NF-kB signaling by targeting SOCS-1 and SIRT1, accelerating tissue injury and inflammation (Jiao et al., 2021). Furthermore, Li et al. analyzed the overall expression of miRNA in renal exosomes from a mouse model of acute kidney injury induced by LPS. They found that these EVs were enriched with miR-19b-3p, which played a role in the interaction between macrophages and tubular epithelial cells. This interaction promoted the polarization of macrophages into M1 macrophages by inhibiting the expression of SOCS-1 and activating the NF-κB signaling pathway. As a result. This ultimately resulted in inflammation in the tubulointerstitial region (Lv et al., 2020). Notably, tumor-derived exosomes containing miR-19b-3p have been found to promote M2 macrophage differentiation and the secretion of exosomal LINC00273. This process ultimately enhances the metastatic potential of lung adenocarcinoma (Chen J. et al., 2021). These findings suggest that EV-miRNAs derived from different cell types can have contrasting effects; however, the underlying rationale for this apparent contradiction remains unclear.

### 3.2 Programmed cell death

Cell death, also referred to as programmed cell death (PCD), is regulated by specific genes within cells. This process is essential for the proper growth and maintenance of homeostasis in multicellular organisms. Currently, researchers have identified at least five primary forms of PCD, namely apoptosis, autophagy, necroptosis, pyroptosis, and ferroptosis (Van der Poll et al., 2021). Much work has demonstrated that the dysregulation of PCD occurs in both immune and non-immune cells in sepsis (Bahar et al., 2018; Bedoui et al., 2020; Zhang et al., 2022b). Emerging evidence suggests that the deregulation of apoptosis mediated by EV-miRNAs plays a significant role in sepsis. Recently, Sun et al. reported that hsa-miR-1262, one of the most highly enriched miRNAs in EVs isolated from serum, is internalized by cardiomyocyte cells. It functions as an inhibitor of the SLC2A1 gene, promoting cell death and inhibiting glycolysis in those cells (Sun F. et al., 2022). Furthermore, a study by another research group has shown that miRNAs isolated from serum can be selectively packaged into EVs and can circulate into cardiomyocyte cells. Within these cells, these miRNAs trigger NLRP3-mediated pyroptosis by binding to HMBOX1, which in turn activates the NF-κB-dependent signaling pathway (Tu et al., 2022). Furthermore, Juan et al. found that the expression of miR-93-5p showed the greatest difference in EVs extracted from different phenotypes of macrophages, with a significant increase in the M2-derived EVs. EVs derived from M2 inhibited pyroptosis in renal tubular epithelial cells, whereas those derived from M1 exacerbated cell damage. MiRNA-93-5p was shown to inhibit the pyroptosis pathway in renal tubular epithelial cells by directly targeting TXNIP, a key molecule for NLRP3 inflammasome activation (Juan et al., 2021). Moreover, as previously described, miR-30d-5p has been shown to trigger inflammatory responses both *in vivo* and *in vitro* while enhancing macrophage pyroptosis. This may provide new insight into the mechanisms underlying the progression of sepsis-induced lung injury (Jiao et al., 2021). Furthermore, a recent study found that miR-210-3p preferentially targets the ATG7 gene in BEAS-2B lung epithelial cells via exosomal trafficking during sepsis-induced acute lung injury, regulating autophagy activation and inflammation in lung epithelial cells (Li et al., 2021).

### 3.3 Endothelial dysfunction and microcirculatory alterations

The impairment of vascular endothelial cells and the disruption of microcirculation are the primary drivers of organ dysfunction in sepsis. This may lead to the emergence of acute respiratory distress syndrome, acute kidney failure, and potentially death (Koh et al., 2010; Darwish and Liles, 2013). During sepsis, the release of numerous inflammatory mediators can induce significant endothelial cell damage, resulting in increased gaps between cells and increased permeability of small blood vessels. Therefore, plasma, red blood cells, white blood cells, and platelets may leak from the blood vessels into the surrounding tissues (Hernandez et al., 2013; Vincent et al., 2021). A growing body of evidence indicates that EVs play a key role in endothelial cell injury during sepsis, with miRNAs contained within EVs emerging as key mediators. In a study conducted by Min Gao et al., it was observed that miR-206-3p, miR-1b, and miR-1-3p were significantly upregulated in EVs isolated from sepsis patients. These miRNAs primarily target the extracellular matrix-receptor interaction pathway and gap junction communication processes. Notably, the expression level of miR-1-3p is the highest compared to other miRNAs and can be taken up by endothelial cells through EVs. MiR-1-3p induces endothelial cell dysfunction by downregulating the SERP1 gene (Gao et al., 2021). Furthermore, the interaction between innate immune and structural cells through the EVs can aggravate inflammation and disrupt the structural barrier (Yuan et al., 2018; Juan et al., 2021). Yuan et al. demonstrated that EVs can regulate the expression of tight junction proteins in bronchial epithelial cells. Specifically, EVs produced from LPS-activated macrophages have been found to significantly reduce the expression of these proteins. This alteration impacts the integrity of the structural epithelial barrier (Yuan et al., 2018). Furthermore, sepsis can induce alterations in microcirculation. Reggiori et al. utilized a laser-assisted rotary cell detector to evaluate microcirculation in patients within the intensive care unit and healthy control group. Their findings showed that severely sick individuals with sepsis had significant alterations in the flow properties of their red blood cells (Reggiori et al., 2009). The reduced deformability of red blood cells and changes in the flow properties can hinder the delivery of oxygen to tissues, potentially leading to organ failure (De Backer et al., 2007; Fernandes, 2009). Interestingly, Subramani et al. found that compared to pre-surgical samples, or the sample from sham-operated animals, the deformability of red blood cells was significantly decreased. They further discovered that EVs isolated from plasma in the two mouse groups exhibited distinct miRNA characteristics. Researchers also found that EVs from CLP-induced mice reduced red blood cell deformability more than those from sham-operated mice. However, the specific functions of these miRNAs in red blood cells and the underlying mechanisms of miRNA changes in CLP-induced mice are still to be fully understood (Subramani et al., 2018).

Conversely, numerous studies have demonstrated that endothelial cells can internalize EVs from various sources to prevent early sepsis-induced endothelium damage and vascular inflammation. One study reported that septic platelets transfer miR-223 into endothelial cells via microparticles, which more effectively downregulated the expression of intercellular adhesion molecule-1 compared to controls. This reduction may decrease white blood cell adhesion and help alleviate excessive vascular inflammation associated with sepsis (Szilágyi et al., 2021). In addition, EV-miR-126 has been shown to stabilize the endothelium during sepsis and modulate sepsis-related organ failure (Zhou Y. et al., 2018; Zhang X. et al., 2020; Zou et al., 2022). However, the role and mechanism of EV-miRNA in endothelial dysfunction and circulatory dysfunction during sepsis have not been fully elucidated, necessitating further research support.

### 3.4 Multiple inflammatory signaling pathways

**FIGURE 3:**
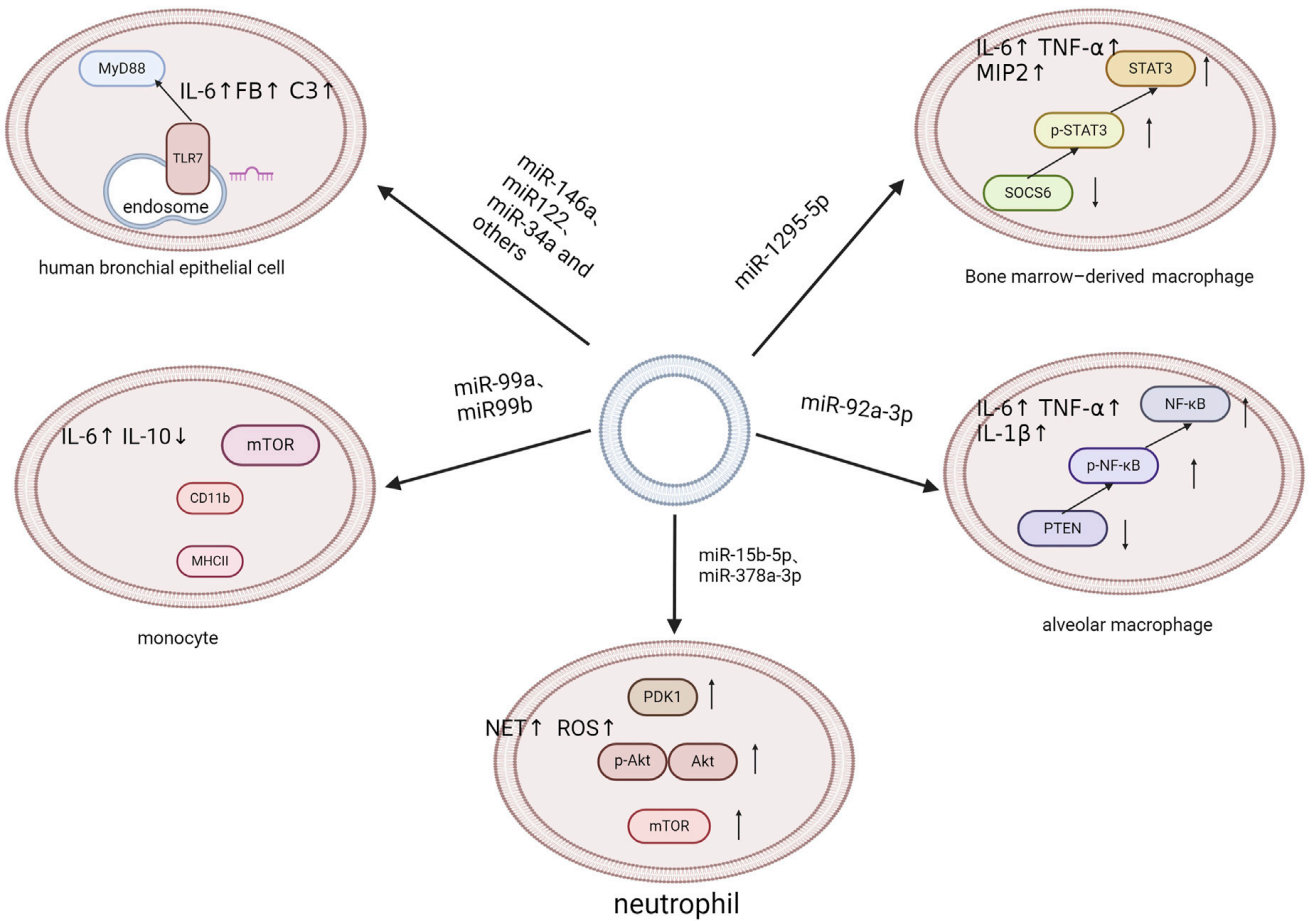
Multiple inflammatory signaling pathways.

A defining characteristic of sepsis is the systemic inflammatory response, which cannot be attributed to a singular causal agent. Instead, it results from a complex interplay of multiple systemic responses. Infection is one of the key causative factors in sepsis that triggers a complex pathophysiological response, encompassing both pro- and anti-inflammatory pathways as well as many non-immune mechanisms (Deutschman and Tracey, 2014). An increasing number of studies have shown that EVs and EV-miRNAs play important roles in the development and progression of sepsis (Roers et al., 2016; Kalluri and LeBleu, 2020). On one hand, EVs have the potential to transport cytokines, extracellular RNAs, and specific proteins between cells. This can lead to inflammatory responses and act as damage-associated molecular patterns (DAMPs) in sepsis (Anand et al., 2010; Collett et al., 2018; Hardy et al., 2019; Li et al., 2020). On the other hand, inflammation also affects the release and internalization of EVs, which can occasionally have an anti-inflammatory effect (Gao et al., 2019). The regulatory function of EV-miRNAs in both anti-inflammatory and pro-inflammatory responses has been increasingly clarified. J Xu et al. demonstrated a correlation between the elevated production of cytokines and the EV delivery of eight specific miRNAs, including miR-34a, miR-122, miR-126-3p, miR-146a-5p, miR-145-5p, miR-26a-5p, miR-150-5p, and miR-181a-5p. These miRNAs regulate the Toll-like receptor 7/MyD88 signaling pathway, which in turn triggers the synthesis of cytokines and complement components, such as complement component 3 and factor B (FB) (Xu et al., 2018). Ma et al. found that miR-1298-5p enhanced inflammation in BEAS-2B cell by suppressing SOCS6 and modulating STAT3 signaling. This finding greatly revealed the mechanism of EVs in sepsis (Ma et al., 2021). Fitzpatrick G and colleagues have identified that EVs isolated from human aortic endothelial cells infected with *Staphylococcus aureus* are enriched with miR-99a and miR-99b. These EVs exacerbate the inflammatory response in sepsis by promoting pro-inflammatory activation of monocytes via inhibition of the mechanistic target of rapamycin (mTOR) (Fitzpatrick et al., 2022). Similarly, Liu et al. proposed that alveolar epithelial cells deliver EVs enriched in miR-92a-3p directly to alveolar macrophages, initiating lung inflammation. This process is associated with activation of the NF-κB pathway and downregulation of phosphatase and tensin homolog (PTEN) expression (Liu F. et al., 2021). Furthermore, Yang et al. demonstrated that platelet-derived EVs containing miR-15b-5p and miR-378a-3p, promote the formation of neutrophil extracellular traps (NETs). NETs are products of the inflammatory response and significant contributors to endothelial damage in the pathophysiology of sepsis. This is mediated by the negative regulation of the Akt/mTOR signaling pathway, which is achieved by suppressing the expression of 3-phosphoinositide-dependent protein kinase-1 (PDK1) (Jiao et al., 2020) (See Figure 3).

As previously mentioned, EVs obtained from mice and individuals with sepsis have been found to have harmful impacts. EV-miRNAs are involved in the progression by regulating macrophage polarization, apoptosis, endothelial cell damage, and various inflammatory pathways. However, other studies have reported that EVs produced from septic mice and patients can protect against infection. For instance, Deng et al. observed that EVs isolated from the plasma of sepsis patients could increase the survival rate of septic mice and exert beneficial effects on T lymphocytes. These effects are mediated through the presence of EV-enriched hsa-miR-7-5p, which specifically targets the mRNA and protein levels of apoptosis-related genes (Deng et al., 2019). EVs derived from the gut of septic mice reduce TNF-α and IL-17A levels in inflamed mucosal tissues, potentially due to the presence of various miRNAs within these EVs (Appiah et al., 2020). The complex characteristics of EVs in sepsis are emphasized by their dual functions, indicating that they could serve as both diagnostic indicators and target points for therapy efforts. Additional investigation is required to completely understand the processes behind these effects and utilize their healing capabilities in cases of sepsis.

## 4 EV-miRNAs in the treatment of sepsis

EVs are characterized by low immunogenicity, low toxicity, high biocompatibility, the ability to traverse biological barriers, and resistance to lysosomal degradation. Therefore, EVs are increasingly becoming the focus of sepsis treatment. Multiple preclinical studies have reported that EV administration significantly reduces tissue inflammation and remodeling, improves pathogen clearance, and reduces morbidity and mortality in multiple preclinical models of sepsis (Yang et al., 2023). EVs have varying regulatory impacts on the cells they target. Here, we summarized the therapeutic effect of EVs in sepsis on different sources (Table 3).

**TABLE 3:** The effect of EV-miRNAs in sepsis on different sources.

Sample source	miRNA	Sample source	Target cell or organ	Related mechanism	Ref.
MSC	miR-223	MSC	Cardiomyocyte	Inhibit the expression of SEMA3A and STAT3, which reduces the inflammatory response and cell death	Wang et al. (2015)
	miR-146a	βMSCs	Macrophages	Promote M2 polarization through alterations in paracrine activity	Song et al. (2017)
	miR-21	βMSCs	Macrophages	Promote macrophage polarization to M2-like phenotype *in vivo* and *in vitro* by targeting *PDCD4*	Yao et al. (2021)
	miR-146b	human umbilical cord mesenchymal stem cell	Renal tubular cells	Regulate NF-κB activity by targeting *IRAK1*	Zhang et al. (2020a)
	miR-27b	MSC	Macrophages	Downregulate JMJD3 and inactivate the NF-κB signaling pathway	Sun et al. (2021)
	miR-17	MSC	Macrophages	Inhibit inflammation and apoptosis by targeting *BRD4/EZH2/TRAIL* axis	Su et al. (2021)
	miR-141	BMSC	Myocardial tissues	Alleviate sepsis-induced myocardial injury by targeting *PTEN* and activating b-catenin	Pei et al. (2021)
	miR-191	BMSC	Macrophages	Target the 3′-UTR of the DAPK1 mRNA and therefore suppress its translation	Liu et al. (2022b)
	miR-26a-5p	MSC	Murine hepatocytes	Inhibit anti-oxidant system by targeting MALAT-1	Cai et al. (2023)
	miR-125b-5p	BMSC	Macrophages	Downregulate STAT3, thereby inhibiting macrophage pyroptosis and downregulating the expression of inflammatory factors	Tao et al. (2023)
	miR-125b-5p	Human-induced pluripotent stem cells	Alveolar macrophages	Suppress NF-κB Signaling in Macrophages by Targeting TRAF6	Peng et al. (2023)
	miR-125b-5p	Adipose-derived stem cells	Pulmonary microvascular endothelial cells	Alleviate pulmonary microvascular endothelial cells ferroptosis and the inflammation via Keap1/Nrf2/GPX4 in sepsis lung injury	Shen et al. (2023)
	miR-21a-5p	MSC	Macrophages	Inhibit the effects of PDCD4 to lead to M2 polarization or downregulated TLR4 and PDCD4 expression	Niu et al. (2023)
	miR-127-5p	BMSC	Lung	Inhibit neutrophil extracellular trap formation by targeting CD64	Zheng et al. (2023)
	miR-150-5p	Human adipose mesenchymal stem cells	Macrophages	Promote the M2 polarization of macrophages via inactivation of the HMGA2-dependent MAPK pathway	Zhao et al. (2024)
	miR-150-3p	Proinflammatory bone marrow mesenchymal Stem	Alveolar macrophages	Suppresses proinflammatory polarization and promoted anti-inflammatory polarization of alveolar by targeting *INHBA*	Liang et al. (2023)
Endothelial progenitor cells	miR-126-5p and 3p	EPC	Endothelial cells	Suppress LPS-induced HMGB1 and VCAM1 levels	Zhou et al. (2018b)
	miR-126-5p and 3p	EPC	Airway epithelial cells	Inhibit *PIK3R2* and *HMGB1* and increasing the levels of tight junction proteins	Zhou et al. (2019)
	miR-93-5p	EPC	HK2 cells	Silence of KDM6B activated H3K27me3 to inhibit TNF-α activation	He et al. (2020)
	miR-21-5p	EPC	Renal tissues	Alleviate the inflammatory and oxidative stress responses and reduce apoptosis in CLP rat by targeting *RUNX1*	Zhang et al. (2021)
	miR-382-3p	EPC	Murine lung, kidney and liver tissues	Inactivate IκBα/NFκB signaling pathway and alleviated the immune suppression by targeting BTRC	Liu et al. (2022a)
Blood	miR-21	Serum	Mouse tubular epithelial cells	Suppress the activation NF-κB and notch signaling, reducing M1 macrophage polarization	Pan et al. (2019)
	miR-145	Blood	BEAS-2B cells	Partially suppresses *TGFBR2/Smad3* signaling	Cao et al. (2019)
	miR-126	Blood	Endothelial cells	Inhibit the mRNA of *VCAM-1* and suppress adhesion molecule expression	Harris et al. (2008), Zhang et al. (2020b)
	miR-142-5p	Serum	Lung tissues	Inhibit *PTEN* and activation of the PI3K/Akt signaling pathway	Zhu et al. (2022)
Specific cell or tissue	miR-24-3p	M2 macrophages	Myocardial tissue	Reduce cardiomyocyte apoptosis by targeting Tnfsf10	Sun et al. (2022b)
	miR-125b-5p	Endothelial cells	Lung tissues	Suppress VEGF level and repressing apoptosis in lung tissue by inhibiting TOP2A expression	Jiang et al. (2021)
	miR-221-3p, miR-221-5p, miR-155-5p, miR-222-5p	Endothelial cells	Cardiomyocytes	Alleviate the apoptosis of cardiomyocytes and reduced the oxidative stress	Cao et al. (2021)
	miR-148a-3p, miR-1a-3p and miR-143-3p	Normal tissues	Macrophages	Limits LPS induced cytokine storm and regulate the function of macrophages	Ge et al. (2023)
	miR-182-5p	Panax ginseng root	Alveolar epithelial cells	Regulate mitochondrial function by targeting NOX4/Drp-1/NLRP3 signal pathway	Ma et al. (2023)

Abbreviation: Sema3A, Semaphorin 3A; PDCD4, Programmed Cell Death Protein 4; CD64, Cluster of Differentiation 64; INHBA, Inhibin Subunit Beta A; HMGB1, High Mobility Group Box 1; VCAM1, Vascular Cell Adhesion Molecule 1; PIK3R2, phosphoinositol-3, kinase regulatory subunit 2; KDM6B, Lysine Demethylase 6B; H3K27me3, Histone 3 Lysine 27 Trimethylation; RUNX1, Runt-related Transcription Factor 1; BTRC, Beta-Transducin Repeat Containing E3 Ubiquitin Protein Ligase; TGFBR2, Transforming Growth Factor Beta Receptor 2; PTEN, phosphatase and tensin homolog, Tnfsf10, Tumor Necrosis Factor Ligand Superfamily Member 10; VEGF, vascular endothelial growth factor; TOP2A, DNA, Topoisomerase II, alpha; NOX4, NADPH, Oxidase 4; Drp-1, Dynamin-Related Protein 1.

### 4.1 Mesenchymal stem cell-derived extracellular vesicles

Multiple studies have shown that EVs derived from stem cells can improve the healing and regeneration of damaged tissue. Additionally, these EVs can reduce inflammation and provide protective effect in different inflammatory conditions, such as sepsis and acute respiratory distress syndrome (Bai et al., 2020; Yu et al., 2020; Shen et al., 2023). Mesenchymal stem cells (MSC) are important members of the stem cell family, and research on sepsis treatment focuses on MSCs, which are mainly derived from bone marrow, fat, and umbilical cord. More than one study has demonstrated that EVs derived from bone marrow mesenchymal stem cells (BMSCs) effectively deliver anti-inflammatory miRNAs, thereby mitigating organ damage. Pei et al. utilized BMSC-derived EVs to observe the impact on myocardial injury in a CLP animal model. BMSCs-derived EVs delivered miR-141, which decreased PTEN expression and increased β-catenin activity. This reduced CLP-induced myocardial dysfunction, inflammatory infiltration, and cell death (Pei et al., 2021). Moreover, EVs containing miR-125b-5p derived from BMSCs downregulate the level of STAT3 and p-STAT3, which reduces lung damage by inhibiting macrophage pyroptosis (Tao et al., 2023). Notably, BMSC-derived EVs can mitigate organ damage induced by sepsis even in a proinflammatory setting. Liang et al. pretreated MSCs with IL-1β (βMSCs) and found that βMSCs-derived EVs effectively increased M2 polarization and protected mice from sepsis. This effect is associated with the upregulation of miR-146a expression and its packaging within EVs (Song et al., 2017). Similarly, Liang et al. also found that miR-150-3p is increasing in EVs isolated from BMSCs following LPS stimulation. This regulated the polarization of macrophages by targeting INHBA, a factor that promotes the suppression of inflammation during acute lung damage (Liang et al., 2023). In addition, Zhang et al. found that human umbilical cord mesenchymal stem cell-derived EVs (HucMSC-EVs) are a useful tool for the repair of sepsis-associated acute kidney injury (AKI). HucMSC-EVs reduce NF-κB activity by reducing the expression of IRAK1 through increasing miR-146b levels. This mechanism ultimately alleviates sepsis-associated acute kidney injury (AKI) (Zhang R. et al., 2020). Notably, both studies observed the protective role of miR-125b-5p in stem cell-derived EVs in sepsis almost simultaneously. Shen et al. reported that miR-125b-5p, present in EVs produced from adipose stem cells, mitigates ferroptosis in pulmonary microvascular endothelial cells by modulating the expression of the Keap1/Nrf2/GPX4 pathway in sepsis-induced lung damage (Shen et al., 2023). Peng et al. differentiated induced pluripotent stem cells (iPSCs) into mesenchymal stem cells (iMSCs) and subsequently extracted small extracellular vesicles (iMSC-sEV) from them. They found that iMSC-sEV has anti-inflammatory capabilities in a model of septic lung damage and attenuates LPS-induced inflammatory response in alveolar macrophages. Higher level of miR-125b-5p in iMSC-sEV targets TRAF6 and inhibits macrophage NF-κB signaling (Peng et al., 2023).

### 4.2 Endothelial progenitor cell-derived extracellular vesicles

Endothelial progenitor cells (EPCs) are another type of pluripotent stem cell that is produced in the bone marrow. EPCs-derived EVs have been utilized in various diseases owing to their superior reparative and angiogenic properties compared to EPCs. Additionally, EVs are less likely to cause allergic reactions. This is also applied to sepsis (Iwaguro et al., 2002; Li et al., 2016; Chen et al., 2022). Administration of EPC-derived EVs has been shown to effectively improve survival rates in septic mice. Further studies revealed that the EVs containing miR-126 suppressed the levels of HMGB1 and VCAM1 in human microvascular endothelial cells induced by LPS. Furthermore, these EVs enhanced the expression of tight junction proteins in small airway epithelial cells, which improves vascular integrity and restoration of pairs of alveolar epithelial barrier function (Zhou Y. et al., 2018; 2019). Similarly, He et al. found that EPCs-derived EVs carrying miR-93-5p played a protective effect on septic kidney injury. This protection was achieved by suppressing the activity of KDM6B and preventing the activation of TNF-α, which reduced inflammation and vascular leakage (He et al., 2020). Furthermore, Zhang et al. discovered that there was an upregulation of the expression of miR-21-5p in EPCs-derived EVs. This upregulation enhanced renal function, mitigated renal tissue damage, reduced the inflammatory response in the bloodstream, and decreased oxidative stress and apoptosis in renal tissues (Zhang et al., 2021). In addition, Liu et al. found that EPCs-derived EVs loaded with miR-382-3p were able to increase the number of lymphocytes, the concentration of Th1 cells, and the ratio of Th1 cells in septic mice. This was achieved by targeting beta-transducin repeat containing E3 ubiquitin-protein ligase (BTRC), which enhanced the immune response and reduced the bacterial load in septic mice (Liu et al., 2022a). These findings suggest the potential of EPC-derived EVs in treating sepsis-induced immunosuppression.

### 4.3 Extracellular vesicles isolated from blood

EVs present in the blood are derived from various sources, including red blood cells, platelets, white blood cells, endothelial cells, and diverse organ cells. The types and content of EVs can vary significantly depending on physiological and pathological conditions. These vesicles play a role in the pathophysiological mechanisms of sepsis by facilitating communication between tissues and organs. Cao et al. found that miR-145 was one of the most downregulated miRNAs in EVs isolated from sepsis patient blood samples. Subsequently, they demonstrated that upregulation of miR-145 was able to reverse LPS-induced inflammation and sepsis-induced lung damage both *in vivo* and *in vitro*. EV-miR-145 reduced sepsis-induced lung injury by inhibiting the TGF-β signaling pathway, which regulates epithelial integrity by modulating phenotypic plasticity and proliferation of epithelial progenitor cells and interactions with other cell types (Cao et al., 2019; Massagué and Sheppard, 2023). Furthermore, research has demonstrated that remote ischemic preconditioning (RIPC) exerts a beneficial effect on organ injury in sepsis. Pan et al. found that kidney injury was less severe in mice with septic kidney injury following RIPC. Ischemic preconditioning of skeletal muscle increased EV miR-21 expression in the serum and protect against septic AKI by suppressing the PDCD-4/NF-κB pathway and PTEN signaling (Pan et al., 2019). Interestingly, miR-142-5p carried by EVs in the serum was found to be elevated in septic acute lung injury (ALI) rats after RIPC. This elevation suppressed septic ALI by reducing PTEN expression and activating the PI3K/Akt pathway (Zhu et al., 2022). These results suggest that RIPC may play an important role in protecting against organ dysfunction in sepsis by transferring EV-miRNAs, showing potential as a therapeutic approach. Recently, a study investigating the effects of electroacupuncture (EA) treatment on sepsis utilized miRNA sequencing and bioinformatics to analyze EVs in the serum of both a normal control group and an EA treatment group. Compared with the normal group, EVs in the EA treatment-group had 53 differential miRNAs, of which 40 were upregulated and 13 were downregulated. These EV-miRNAs may regulate several important genes (e.g., HSP90AA1, GRB2, EGFR) and signaling pathways (e.g., MAPK, TNF, JAK-STAT) related to inflammation, immune regulation, metabolism, and cell proliferation and apoptosis (Zhang et al., 2023). Furthermore, a thorough proteome and transcriptome investigation of sepsis serum EVs by Li et al. They found a functional network linking vitamin and folate metabolic responses to EVs in serum carrying differently miRNA-targeted genes. Notably, they pretreated a CLP mouse model with EVs in serum from septic animals, which reduced tissue damage and affected vitamin digestion and absorption metabolites. This suggests that EVs in serum play a regulatory role in vitamin metabolism during sepsis. The application of EVs derived from the serum of septic patients as a treatment for sepsis may represent a promising avenue for future research (Li et al., 2022). Thus, EVs can convey specific information and transport it to the lesion site, acting as an anti-inflammatory in sepsis. The interaction of mRNA targets among multiple miRNAs constitutes a key component of the pathophysiology of sepsis. Further exploration of the interaction of multiple miRNAs will benefit more patients with sepsis.

### 4.4 Specific cell or tissue derived extracellular vesicles

Increasing evidence suggests that EVs isolated from certain cells or tissues can be involved in the progression or recovery of sepsis. Michael et al. administered EVs extracted from the intestines of septic mice to treat mice with DSS-induced colitis. They found a reduction in intestinal TNF-α and IL-17A levels, leading to a significant improvement in colitis-related intestinal inflammation. These changes may be related to 51 altered miRNAs in EVs (Appiah et al., 2020). Cao et al. found that EVs produced by the appropriate concentration of LPS-stimulated endothelial cells have protective effects on neonatal rat cardiomyocytes, which was related to changes in the abundance of multiple miRNAs in EVs (Cao et al., 2021). Additionally, Jiang et al. utilized EVs derived from vascular endothelial cells to treat ALI mice and found a reduction in the extent of lung injury in these animals. EVs effectively deliver miRNAs to the site of lung injury, inhibit lung inflammation, and regulate VEGF expression in tissues and serum by targeting topoisomerase IIα (Jiang et al., 2021). Notably, Ge et al. discovered that EVs derived from normal tissues (including murine heart, lung, liver, and kidney) can mitigate the cytokine storm induced by LPS in macrophages. The fact that EVs carry numerous miRNAs, such as miR-148a- 3p, miR-1a-3p, and miR-143-3p, which may reduce macrophages’ excessive inflammatory response to LPS through a variety of pathways, including STAT3, P65, and SAPK/JNK (Ge et al., 2023). However, further experiments are necessary to elucidate the mechanisms by which EV-miRNAs reduce inflammation. Additionally, studies have shown that M2 macrophage-derived EVs have been proven to reduce inflammation (Saqib et al., 2018; Dai et al., 2020). Sun et al. found that M2 macrophage-derived EVs are rich in miR-24-3p and significantly inhibit cardiomyocyte apoptosis, thereby improving myocardial damage by suppressing tumor necrosis factor-associated apoptosis-inducing ligands (Tnfsf10) (Sun X. et al., 2022). Furthermore, more evidence suggests that plant-derived EVs are crucial in regulating the immune system, combating tumors, promoting regeneration, and treating inflammatory diseases (Xu et al., 2021; Mu et al., 2023). Ma et al. loaded miR-182-5p into EVs derived from Panax ginseng root, which were coated with human neutrophil cell membrane. This study demonstrated that EVs derived from Panax ginseng root can serve as an efficient and safe nanocarrier for delivering active miRNAs into alveolar epithelial cells, thereby inhibiting inflammatory damage *in vivo* and *in vitro* by regulating the NOX4/Drp-1/NLRP3 pathway (Ma et al., 2023).

## 5 Extracellular vesicle miRNA-Based prognosis and diagnosis of sepsis

Although IL-6, procalcitonin, and C-reactive protein are commonly utilized biomarkers for assessing infection and hypothesizing potential causes, they primarily reflect inflammation levels and exhibit limited utility in early detection and predicting outcomes. The potential of circulating miRNAs, particularly EV-enriched miRNAs, as biomarkers for sepsis has emerged, given that individuals with sepsis exhibit altered levels of circulating miRNAs (Ying, et al., 2017; Kadri et al., 2018). Several studies indicate that EV-miRNAs exhibit significant differential expression in patients with sepsis compared to healthy volunteers (Real et al., 2018; Shin et al., 2023). Among these, one study suggests that altered levels of miR-15a, miR-27a, and miR-34a in plasma may influence endothelial dysfunction and the development of shock, serving as useful prognostic biomarkers for the risk stratification of patients with severe sepsis. These miRNAs may derive from EVs isolated from human endothelial progenitor cell, but this study failed to demonstrate this hypothesis (Goodwin et al., 2015). Moreover, Reithmair et al. compared miRNAs levels across total serum, serum EVs, and blood cells (including white blood cells, red blood cells, and platelets). They found that miR-125b-5p and miR-26b-5p were regulated in both EVs and serum, whereas miR-199b-5p was identified as a potential biomarker for sepsis and septic shock within the blood cellular compartment. Despite the current lack of clinical evidence supporting the role of these miRNAs in sepsis, the differential expression of various miRNAs across different compartments may serve as potential biomarkers for diagnosing sepsis (Reithmair et al., 2017). Moreover, a study investigated the expression of circulating miRNAs in healthy individuals, patients with community-acquired pneumonia (CAP), and patients with sepsis. The study showed that miR-1246 increased significantly with overall disease severity, while miR-193a-5p and miR-542-3p distinguished patients with infectious diseases (CAP or sepsis) from healthy individuals (Hermann et al., 2020). Moreover, another study found that miR-181a-5p and miR-23b-3p were differentially expressed in circulating EVs earlier than creatinine elevation in sepsis-induced AKI in rats. These variations in miRNA expression may serve as valuable tools for the early identification of sepsis-induced AKI and for differentiating it from other causes of AKI (Da-Silva et al., 2022). Additionally, the brain tissue, cerebrospinal fluid, and plasma of rats with sepsis-associated encephalopathy exhibited significant differences and correlations in four specific miRNAs: miR-127-3p, miR-423-3p, miR-378b, and miR-106-3p. These findings suggest that detecting EV-miRNAs may enable the capture of brain histopathological changes during sepsis-associated encephalopathy (Xiao et al., 2024). A recent study attempted to investigate the relationship between EV-miRNAs and the prognosis of sepsis. They screened 25 EV-miRNAs in sepsis serum which are significantly different from healthy individuals and found that lower expression of miR-335-5p, miR-301a-3p, hsa-let-7f-5p, and miR-331-3p was significantly associated with in-hospital and 90-day mortality, demonstrating their potential as early prognostic tools for sepsis (Shin et al., 2023). A recent study also showed that sEV MIR497HG, miR-195, and miR-497 change periodically with the progression of sepsis, which is associated with 28-day mortality in patients with sepsis (Liu et al., 2023).

## 6 EV-miRNA as a diagnostic and therapeutic tool for sepsis: prospects and challenges

As previously mentioned, EV-miRNAs offer a non-invasive approach for early diagnosis and progressive prognosis of sepsis. Numerous researchers are actively exploring the use of EV detection methods for disease diagnosis. For example, McKiernan et al. conducted two prospective experiments to verify whether a novel urine exosome gene expression test can predict high-grade prostate cancer in patients with ambiguous prostate-specific antigen test results. This detection method can improve the differential efficiency of high-grade prostate cancer, low-grade prostate cancer, and benign prostate lesions from 0.63 to 0.73 in 1,500 samples from 22 centers (McKiernan et al., 2016; 2018). Sepsis is characterized by rapid progression, potentially evolving from a localized infection to a systemic inflammatory response within hours to days, ultimately resulting in multiple organ failure and death. Consequently, the development of EV-miRNA reagents holds significant promise for the early diagnosis of sepsis. Nonetheless, the application of EV-miRNAs for the prognosis and diagnosis of sepsis currently encounters significant challenges. First, despite recent advancements in EV purification methods, a rapid, simple, stable, and clinically applicable EV purification technique for clinical specimens remains unavailable, severely limiting the clinical application of EVs. Second, the preservation, purification, disruption, and identification of EV specimens necessitate the development of comprehensive standardized protocols and quality control measures, as there are currently no widely recognized standards or methodologies (Romanò et al., 2024). Additionally, one limitation is that the sample size used to evaluate circulating EV-miRNA as a biomarker for sepsis is still relatively small. Furthermore, these samples often cannot exclude the presence of multiple pre-existing conditions, such as diabetes and hypertension, which may have various effects on EV-miRNAs.

Sepsis is considered a systemic inflammatory response triggered by infection, often accompanied by organ dysfunction. Current treatment strategies primarily focus on controlling the infection and sustaining life. The fourth section discusses the use of EVs derived from MSCs, endothelial progenitor cells, endothelial cells, and normal tissues to inhibit inflammatory responses and reduce organ damage in sepsis models. This serves as an ideal drug delivery system. There is a great promise for EV application based on biologically targeted therapy. However, since most of these studies are preliminary investigations conducted in animal models, there remains a need to translate the findings into clinical applications. Furthermore, any treatment that interacts with underlying biological signaling pathways entails inherent risks. Therefore, rigorous and comprehensive risk and safety analysis is necessary before drug-carrying exosomes can be developed for human trials.

## 7 Discussion

EV-miRNAs in sepsis are currently associated with macrophage polarization, programmed cell death, inflammation, endothelial dysfunction, and organ damage. Moreover, several studies suggest a link between EV-miRNAs, clinical outcomes, and survival prediction. Among these, several notable phenomena warrant discussion. As previously mentioned, Sun et al. found that EVs extracted from the serum of sepsis patients induce cardiomyocyte death and inhibit glycolysis in these cells (Sun F. et al., 2022). However, Jiang et al. found that EVs with elevated miR-155 expression can enhance M1 polarization and pro-inflammatory responses in macrophages by inhibiting SHIP1 and SOCS1 (Jiang et al., 2019). SOCS1 functions as a regulator of metabolic reprogramming. Suppressing SOCS1 may promotes glycolysis and pro-inflammatory responses in sepsis murine bone marrow cells via the STAT3/HIF-1α axis This implies that EV-miRNA may promote the glycolysis of septic macrophages (Alvarez et al., 2017). This intriguing phenomenon has prompted researchers to explore the relationship between EV-miRNA and metabolic reprogramming within the context of sepsis. However, there is currently a lack of experimental data to explain this issue. Similarly, determining whether EV-miRNAs are harmful or beneficial in sepsis remains challenging.

Sepsis is a complex clinical illness with dynamic dysregulation across multiple levels and systems. Its complexity is not only reflected in inflammation and immune responses but also in the interactions of hemodynamics, coagulation, metabolic abnormalities, and multiple organ failure (Abraham and Singer, 2007). This review elucidates the role of EV-miRNA in the pathogenesis of sepsis. Unfortunately, the majority of the EVs analyzed in the study were isolated from inflammatory cells and blood samples. Few studies have investigated the role of EVs in immunosuppressive conditions. This limitation results in a lack of in-depth discussion of our findings. Furthermore, some studies have isolated EVs from mouse or human serum for the treatment of septic animals or to measure biomarker levels (Li et al., 2022). Most of these studies had good clinical outcomes. However, the majority of published trials lack detailed information regarding the precise timing of EV isolation and usage, as well as information on EV dosage. We are currently unable to compare these findings.

The role of EV-miRNA is influenced by the source of the EVs and the specific type of miRNA. This review summarizes the involvement of EVs from MSCs, EPCs, and particular cells in the treatment of sepsis. The function of MSC and EPC is influenced by the donor source (e.g. bone marrow, adipose tissue, umbilical cord), which subsequently leads to significant differences in the types and effects of EV-miRNAs (Rohban and Pieber, 2017). Additionally, many of these studies lack detailed descriptions of the EV dosage, frequency, and method of delivery. This makes it difficult to replicate experimental results and also makes clinical translation of EV-miRNA for sepsis difficult.

The MISEV guidelines emphasize the necessity for standardization of EV isolation methods, EV storage conditions, EV characterization procedures, and quality control. This will help to reduce potential biases and risks while also ensuring the accuracy and reproducibility of research findings (Welsh et al., 2024). Ultracentrifugation, polymer precipitation, and size exclusion chromatography are now widely used separation procedures. Each separation method has limits. For example, ultracentrifugation cannot distinguish between various EV subpopulations and cell detritus. The speed, duration, and radius of centrifugation can significantly impact experimental outcomes. Polymer precipitation is highly susceptible to protein contaminants, resulting in inaccurate outcomes (Konoshenko et al., 2018). Additionally, unstable sample storage temperatures and excessive freeze-thaw cycles can cause EV degradation, thereby affecting experimental outcomes (Jeyaram and Jay, 2018). However, not all of the studies included in our review fully described this information. Consequently, making broad generalizations from the literature necessitates caution. Furthermore, the characteristics of EVs released under different cell-types, disease states, or stimulus conditions exhibit significant variability. This heterogeneity must not be overlooked, as it may result in erroneous generalizations of conclusions.

Finally, in the pathological process of sepsis, EV-miRNA enhances inflammation and multi-organ failure through multiple mechanisms. EV-miRNA has great potential in the treatment of sepsis, early diagnosis, and evaluation of prognosis. In future studies, attention needs to be paid to describing the cellular origin of EV, isolation methods, and elucidating the role EV plays in specific sepsis stages, which will likely advance the clinical application of EV-miRNA for sepsis.
